# Three New Species and a New Record of Arbuscular Mycorrhizal Fungi of the Genus *Acaulospora* Associated with Citrus from South China

**DOI:** 10.3390/jof11050382

**Published:** 2025-05-16

**Authors:** Haisi Huang, Xiaojuan Qin, Yihao Kang, Jie Xu, Pengxiang Shang, Tingsu Chen, Tong Cheng, Jinlian Zhang

**Affiliations:** 1 Microbiology Research Institute, Guangxi Academy of Agricultural Sciences, Nanning 530007, China; huanghs0812@163.com (H.H.); qxj3249302@163.com (X.Q.); jiexu0331@163.com (J.X.); chents001@gxaas.net (T.C.); 2 School of Public Health, Xiamen University, Xiamen 361102, China; kangyh0810@163.com (Y.K.); pxshang@126.com (P.S.); 3 School of Life Sciences, Xiamen University, Xiamen 361102, China; tcheng@xmu.edu.cn

**Keywords:** glomeromycota, arbuscular mycorrhizal fungi, *Acaulospora*, phylogenetics, taxonomy

## Abstract

Arbuscular mycorrhizal (AM) fungi are root symbionts that play an important role in the growth of vascular plants. Four AM fungi, including three new species, *Acaulospora citrusnsis*, *A. guangxiensis*, *A. jiangxiensis*, and a new country record from China, *Acaulospora herrerae*, are reported based on morphological characteristics and molecular phylogenetic analysis. They were isolated and propagated from spores extracted from the rhizosphere soils of citrus. *A*. *citrusnsis* is characterized by forming hyaline to pale yellow globose to subglobose spores of (70.0–)85.0(−100.0) μm in diameter. Spores of *A. guangxiensis* are pale yellow to pale yellowish brown, with spherical to sub-spherical appearance and (103.1–)122.1(–147.1) μm in diameter. Young spores of *A. jiangxiensis* are hyaline, gradually turning pale yellow as they mature, with spherical to sub-spherical appearance and (78.7–)85.6(–90.0) μm in diameter. Spores of *A. herrerae* are hyaline and 86.3–127.2 μm in diameter. Four species have three spore wall layers, and spores form individually in the soil. The phylogenetic tree was constructed and inferred from sequences of 18S-ITS1-5.8S-ITS2-28S datasets by Bayesian inference and maximum likelihood analysis. Voucher specimens are deposited in the Center for Subtropical Arbuscular Mycorrhizal Fungi Culture Collection (CSMC).

## 1. Introduction

Arbuscular mycorrhizal (AM) fungi are important microorganisms within the phylum Glomeromycota. They are widely distributed in soils around the world and form symbiotic relationships with the roots of more than two-thirds of terrestrial plant species [1,2]. AM fungi promote plant growth, increase the absorption of mineral elements, and help the host plant enhance its resistance to pathogens and diseases [3,4]. AM fungi absorb more water for the host through extraradical mycelium and secrete arbuscular mycorrhizal proteins (defined as soil proteins associated with AM fungi) into the soil to stabilize soil aggregates, thereby improving soil water-holding capacity and aeration [5]. Additionally, AM fungi can alleviate heavy metal toxicity by competitively absorbing heavy metal ions from the soil [6,7], while simultaneously improving the soil environment [8,9,10,11]. Therefore, AM fungi are crucial for plant health, survival, and the restoration of native ecosystems, as well as for good soil structure [12].

The biological and functional diversity of AM fungi is crucial to agricultural ecosystems [13]. Citrus is one of the most widely cultivated fruit crops, and the roots of citrus harbor these beneficial fungi [5]. Due to the fewer and shorter root hairs in citrus roots, they are highly dependent on AM fungi. To date, 45 species of fungi have been identified in the rhizosphere of citrus plants, belonging to seven genera: *Acaulospora*, *Entrophospora*, *Gigaspora*, *Glomus*, *Pacispora*, *Sclerocystis*, and *Scutellospora*, which colonize citrus roots and form arbuscular mycorrhizal symbiotic relationships [12]. Studies have shown that different types of AM fungi have significant differences in their effects on citrus [14].

The genus *Acaulospora*, within the phylum Glomeromycota, widely distributed in Brazil, India, Switzerland, and other regions, especially in tropical areas, is one of the most widely distributed genera in the world; in recent years, several species have been introduced [15,16,17,18]. Many studies have highlighted the ability of *Acaulospora* to develop under extreme conditions, such as in high-salinity soils [19,20], as well as in soils contaminated with nickel [21] and arsenic [22]. *Acaulospora* species play an important role in soil use and management, particularly in semi-arid regions [16,17,18,23]. This resistance to various abiotic stresses demonstrates the biotechnological potential of the *Acaulospora* species in agricultural and bioremediation activities. Additionally, *Acaulospora* species have been reported as the second most common genus in disturbed areas, with a high diversity index, primarily distributed in South America [16,24]. The genus *Acaulospora* has been found in 61 countries worldwide, primarily distributed in temperate and tropical regions, with more than two-thirds of the species discovered in Brazil [25]. Continued discovery and description of new species within the genus *Acaulospora* will help maximize their value in biotechnological applications and regional conservation.

With the development of molecular identification techniques, particularly for genes related to AM fungi, including the partial nuclear ribosomal DNA small subunit (18S), the internal transcribed spacer region (ITS1-5.8S-ITS2, abbreviated as ITS), partial large subunit (28S) fragments (18S-ITS-28S), and the largest subunit of the RNA polymerase (RPB1), new approaches and solutions have been provided for the identification of AM fungi [26,27,28]. In recent years, approximately 30 new species of AM fungi have been discovered, most of which are from Europe and Americas, and only four species have been discovered in China: two from the Tibetan Plateau (at an altitude of 2843 m) and two from Mount Fanjing (at an altitude of 1900–2400 m) [26,29,30,31,32,33,34,35,36,37,38,39,40,41,42].

Our morphological and molecular studies have revealed three undescribed species and one previously unreported species of AM fungi within the *Acaulospora* genus associated with citrus. These species are part of the AM fungi community related to citrus in China. Our research team extracted over 1500 individual spores from 90 soil samples collected from 9 representative citrus orchards in Jiangxi and Guangxi provinces, establishing a large number of single-specific AM fungi pot cultures. Four of these cultures form spores typical of the *Acaulospora* genus, including three new species: *Acaulospora citrusnsis*, *Acaulospora guangxiensis*, *Acaulospora jiangxiensis*, and a new record for China: *Acaulospora herrerae*.

The aim of this study is to isolate, describe, and identify the collected AM fungi through a combination of morphological observations and phylogenetic analyses, with the goal of revealing their species diversity and evolutionary history, thus laying the foundation for further ecological and applied research.

## 2. Materials and Methods

### 2.1. Study Site, Soil Sampling, and Fungal Spore Extraction

This study collected rhizosphere soils from both healthy and Huanglongbing-infected citrus trees in 9 representative citrus orchards located in Jiangxi and Guangxi provinces of southern China (Table 1). A total of 90 soil samples were collected. Among these, 68 samples (34 healthy and 34 Huanglongbing-infected) were collected from 5 citrus orchards in Ganzhou, Jiangxi. The citrus varieties studied were Hamlin sweet orange (*C. sinensis* (L.) Osbeck) and Newhall navel orange, both grafted onto trifoliate orange rootstock. Additionally, 22 samples (11 healthy and 11 Huanglongbing-infected) were collected from Fuchuan County in Guangxi. The citrus varieties studied in this region were Newhall navel orange, early-maturing Wenzhou mandarin, and sugar tangerine, all grafted onto trifoliate orange rootstock. Rhizosphere soil samples (500 g) were collected to analyze the AM fungi community. Additionally, AM fungi spores were extracted from 20 g of air-dried soil using the wet sieving method for monosporal isolation and cultivation [42].

### 2.2. Monosporic Cultures and Staining of Mycorrhizal Structures

Monosporic cultures were established with spores isolated from field-collected soil and grown as given by Błaszkowski et al. [43]. *Zea mays* Linn. and *Citrus junos* Siebold ex Tanaka were used as host plants for single-species cultivation, with approximately 50 individual spores of each type planted in the root systems of the host plants. After four months, soil was collected, and spores were extracted using the wet sieving method [42]. Root segments (0.5–1.0 cm in length) were collected and stained using the Quink ink–vinegar solution method to assess root infection [44].

### 2.3. Morphological Analyses of AM Fungi Spores

Spores from single-species cultures of the new fungus were extracted as previously described. After examining at least 100 spores under a microscope, their morphological characteristics, spore wall phenotype, and histochemical properties were determined. The spores were observed in water, lactic acid, PVLG [45], and a mixture of PVLG and Melzer’s reagent (1:1, *v*/*v*) as mounting media [46]. The description of the subcellular structure of the spores was carried out following the method of Błaszkowski et al. [43]. After immersing the fresh specimens in water, the color of the spores was observed using a dissecting microscope, and the color was determined with the INVAM color chart. Photographs were captured with a digital camera (Nikon DS-Ri2, Shinagawa, Japan) mounted on a Nikon Eclipse Ci-L microscope equipped with a Nomarski differential interference contrast optical system. Photographs’ processing was performed using the NIS-Elements version D 4.3 software. Specimens mounted in PVLG and a (1:1) mixture of PVLG and Melzer’s reagent were deposited at the Center for Subtropical Arbuscular Mycorrhizal Fungi Culture Collection (CSMC) (China, Guangxi) in CSMC-GJ-JX-1. Morphological comparison of spores from the seven obtained species of the genus *Acaulospora* was conducted using species descriptions available on the INVAM website (http://fungi.invam.wvu.edu/thefungi/species-descriptions.html, accessed on 1 December 2024) and newly published species descriptions [47,48,49].

### 2.4. DNA Extraction, PCR Amplification, and Sequencing

Genomic DNA was extracted from a single glomerospore. The specific steps are as follows: Fresh spores were sonicated for 10 min and rinsed three times with sterilized distilled water. Subsequently, a single spore was transferred to an Eppendorf tube and ground into a fine powder, to which 10 μL of ultrapure water was added, with 2 μL used for polymerase chain reaction (PCR). Using the AM fungi-specific primers developed by Krüger et al. [27], a DNA fragment of approximately 1545 bp was amplified by nested PCR, covering part of the SSU, the entire ITS, and the variable D1 and D2 regions of the LSU. In the first round of PCR, the primers SSUmAf and LSUmAr were used. In the second round of PCR, 1 μL of the first round PCR product was used as a template, with the primers SSUmCf and LSUmBr. The PCR mix included 0.4 U of AmpliTaq^®^ 360 DNA polymerase (Applied Biosystems, Foster City, CA, USA), 1X AmpliTaq^®^ 360 PCR buffer (Applied Biosystems), 0.2 mM of each dNTP, 0.4 μM of each primer, and 1 μL of the template in a final volume of 25 μL. The cycling parameters for the first PCR were 3 min at 98 °C followed by 35 cycles of 10 s at 98 °C, 30 s at 60 °C, and 1 min at 72 °C. The program was concluded by a final extension phase of 10 min at 72 °C. The cycling parameters for the second PCR were the same as in the first PCR except for the number of cycles (35) and annealing temperature (63 °C). The PCRs were conducted in triplicate. PCR products were checked on 1% agarose gels, and the PCR products with the expected-size bands were purified with the DNA Gel Extraction Kit. The PCR-positive products were cloned into the pGEM-T vector system (Promega, Madison, WI, USA) following the manufacturer’s instructions. The ligated plasmids were transformed into CaCl_2_ competent *Escherichia coli* DH5α cells using a heat-shock approach. The transformed bacteria were plated into LB (Luria-Bertani) medium containing ampicillin (50 μg/mL) and grown overnight at 37 °C. A PCR using the universal M13F and M13R primers was performed directly on bacterial colonies to screen for positive clones. Clones that exhibited fragments with the expected size were sequenced on an Applied Biosystems 3730xl capillary sequencer (IRD, Noumea, New Caledonia) with the BigDye^®^ Terminator v3.1 Cycle Sequencing Kit (Applied Biosystems).

### 2.5. Sequence Alignment and Phylogenetic Analyses

The representative sequences of the 4 obtained species were aligned through BLAST on GenBank (https://blast.ncbi.nlm.nih.gov/Blast.cgi, accessed on 1 January 2025), and a phylogenetic tree was constructed by downloading 57 closely related sequences, using *Sacculospora baltica* and *S*. *felinovii* [25] as the outgroup (Table 2). Maximum Likelihood (ML) and Bayesian Inference (BI) analyses were employed to clarify the phylogenetic positions and relationships of the species. The ML analysis was performed using the MEGA 7.0 program (v1.0.0.0). Sequence alignment was carried out with MUSCLE in MEGA 7 using default parameters to optimize the downloaded sequences. Model prediction was executed with the model function in MEGA 7.0, followed by the Maximum Likelihood analysis. The best-fit evolutionary model of alignment was determined by Modelgenerator (v851) [50]. Bayesian analysis was conducted using software MrBayes v3.2.2. The number of generations was set to 2,000,000, and trees were being sampled every 1000 generations (a total of 2000 trees), and the average standard deviation of split frequencies was below 0.01 [51].

## 3. Results

### 3.1. Phylogenetic Analyses

**Figure 1:**
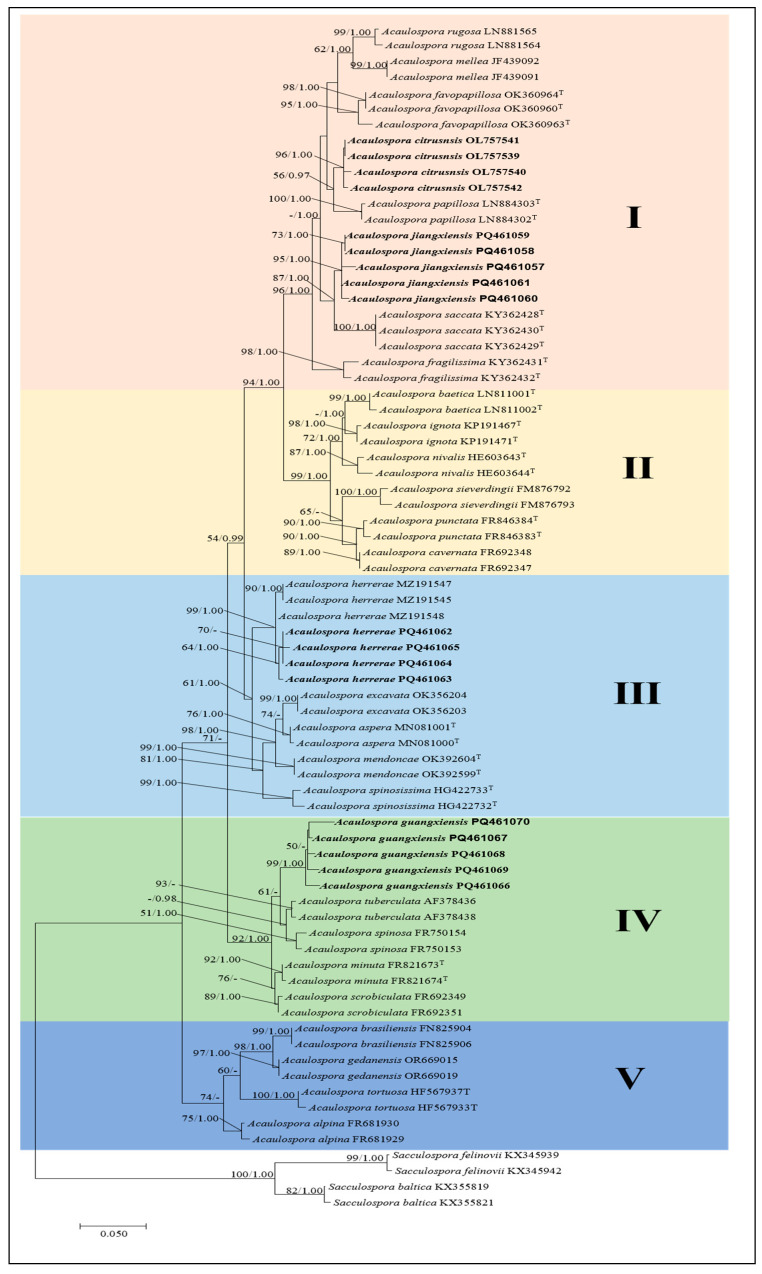
Phylogenetic tree generated from maximum likelihood analyses based on the SSU-ITS-LSU sequences, expressing the relationship of *Acaulospora* species. Maximum likelihood bootstrap support (ML-BS) ≥ 50% (left) and Bayesian posterior probability (BPP) values ≥ 95% (right) are indicated at nodes (MLBS/BPP). *S. baltica* and *S. felinovii* were included as outgroups. Bold names represent new species and new Chinese records. I–V indicate different clades.

Phylogenetic trees were constructed from the sequences of the rDNA region, comprising partial small subunit rRNA gene, the internal transcribed spacers, 5.8S rRNA gene, and the partial large subunit rRNA gene. The obtained trees from ML and BI were similar, and only the ML tree is shown (Figure 1). The dataset contained 75 taxa with *S. baltica* and *S. felinovii* as outgroups for investigating molecular phylogeny. The best model for constructing the Bayesian tree of the datasets was found to be GTR + G, lset nst = 6, Rates = gamma, prset statefreqpr = fixed (0.29577, 0.15604, 0.21257, 0.33563), –In*L* = 12,357.36516. The gamma distribution shape: *α* = 0.37. The average standard deviation of split frequencies of Bayesian’s analysis remained 0.003517. The final ML best model was T92 + G, and the optimization likelihood value was −2892.113. Bootstrap support values with ML greater than 50%, and BPP values greater than 0.95 are given above the nodes (Figure 1).

The phylogenetic analysis revealed that the new species *A. citrusnsis* was closely related to *A. papillosa* from Brazil, supported with a medium bootstrap support value (ML-BS = 56%, BPP = 0.97), indicating a sister relationship between these two species. *A. jiangxiensis* was located in a clade with high statistical support (ML-BS = 87%, BPP = 1.00) with *A. saccate* from New Caledonia. The fine sequences of the *A. jiangxiensis* were clustered in one branch to form a subclade with strong support and designated as *A. jiangxiensis* (ML-BS = 95%, BPP = 1.00). *Acaulospora guangxiensis* was related to *A. tuberculata* and *A. spinosa* clustered together in a low support subclade (ML-BS = 95%, BPP = –). In addition, four sequences of *A. herrerae*, a newly recorded species in China, were clustered in a branch with *A. herrerae* from Peru to form a subclade with high support value (ML-BS = 99%, BPP = 1.00), suggesting that they may be of the same species. Our phylogenetic analyses of SSU–ITS–LSU rDNA sequences of three likely yet unnamed AM fungi and a new record species confirmed our supposition and proved that they belong in the genus *Acaulospora* and indicated their closest species relatives.

### 3.2. Taxonomic Analyses

#### 3.2.1. New Species

**Figure 2:**
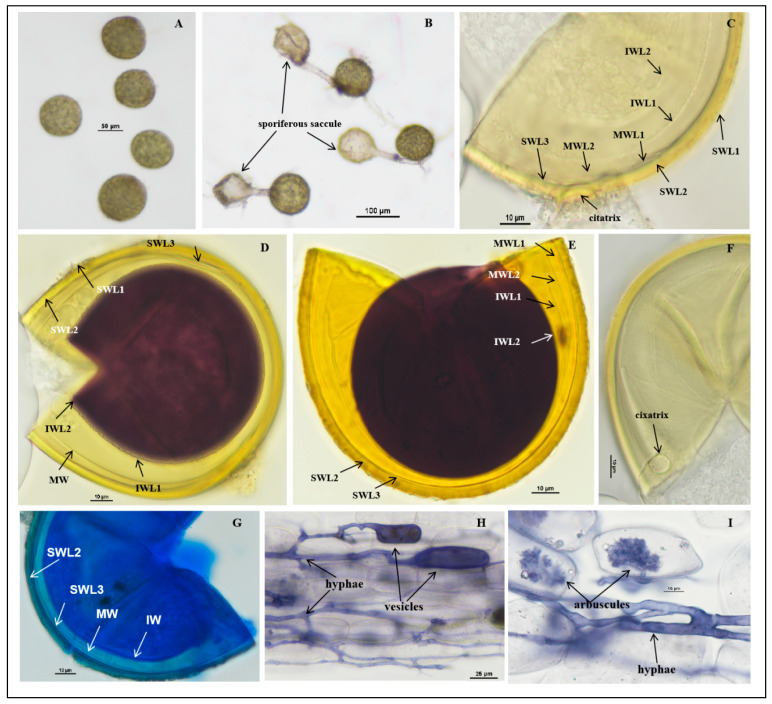
*Acaulospora citrusnsis* spores. (**A**) Intact spores in water; (**B**) intact spores with sporiferous saccule in root organ culture; (**C**–**E**,**G**) spore wall layers: SWL1 hyaline, forming the spore surface, usually absent in mature spores; SWL2 and SW L3 pale yellow to pale yellowish brown, permanent, laminate, and similar color; in some spores, the boundaries between layers are difficult to distinguish. The inner wall hyaline consists of two layers. IWL2 shows a pale pink reaction to Melzer’s reagent; (**F**) cicatrix; (**H**,**I**) mycorrhizal structures of *A. citrusnsis* in roots of *C*. *junos* stained in Quink ink–vinegar solution: hyphae, vesicles, and arbuscules; (**D**) in Melzer’s reagent; (**E**) in PVLG + Melzer’s reagent; (**B**,**F**) in PVLG reagent; (**G**) in laetophenol cotton blue.

*Acaulospora citrusnsis* J.L.Zhang, sp. nov. Figure 2A–I

MycoBank MB 856567

Description: Sporocarps unknown. Spores form singly in soil, borne laterally from the neck of a sporiferous saccule (Figure 2B). At the proximal end of mycelia, there is a sporiferous saccule which is similar in size to the spores. The sporiferous saccule shrinks and becomes empty after the spores mature completely and, usually, shed after wet screening: spores sessile, globose to subglobose, (70.0–)85.0(−100.0) μm in diameter; occasionally elliptic, (72.0–85.0) × (100.0–110.0) µm, hyaline when young, later pale yellow. Spore wall (SW) consists of three layers (L1–L3), SWL1 forming the spore surface, evanescent, hyaline, (0.5–)1.0(–1.2) μm thick, usually with debris attached to it. When not degraded, it is 0.7–1.0 µm thick. With the aging of the spores, it degrades, leaving only residual attachment L2, usually absent in mature spores (Figure 2C–E), dyed dark blue in the lactophenol cotton blue reagent (Figure 2G); SWL2 permanent, pale yellow, pale brownish yellow, laminate, consisting of multiple sublayers of varying thickness, 3.1–4.9 µm thick, no obvious discoloration in Melzer’s reagent and PVLG reagent, lactic acid phenol cotton blue reagent with longer staining time, finally dyed dark blue (Figure 2C–E,G). SWL3 concolorous with SWL2, translucent, tightly adherent to L2, and can be separated from L2 after forced crushing of spores, 1.0–1.5 µm thick. None of these wall layers stains in Melzer’s reagent (Figure 2C–E,G).

The middle wall is bi-layered (MWL1–2) and semi-flexible layers. The layers are commonly tightly adherent and adherent to the inner wall with beadlike particles, hyaline to pale yellow, of almost equal thickness, and together 0.8–1.1 µm thick (Figure 2C–E,G).

The inner wall is with two thin, hyaline layers (IWL1–L2); IWL1 is a ductile wall, 0.5–0.8 µm thick, covered with small granular excrescences about 0.5 µm in diameter, and after spore crushing, usually evenly distributed. IWL2 in PVLG at least 1.0–1.2 μm thick, plastic, turning deep purplish red to brownish red in Melzer’s reagent (Figure 2D,E), dyed blue in lactic acid phenol cotton blue reagent (Figure 2G). Germination orb was not observed.

Mycorrhizal associations: In the field, *A.* *citrusnsis* associated with the rhizosphere of citrus Newhall navel orange, *C. sinensis* (L.) Osbeck, and *C. reticulata* Banco (*Poncirus trifoliata* (L.) Raf.). In Monosporic cultures with the *C. junos* host plant, *A. citrusnsis* formed mycorrhiza with arbuscules, vesicles, and hyphae (Figure 2H,I).

Etymology: *Acaulospora citrusnsis* (Latin), referring to this species was first recorded with citrus plants as its host.

Distribution and habitat: Spores of *A. citrusnsis* were isolated from rhizosphere soil of a citrus orchard from Jiangxi province in South China: lateritic soil and subtropical monsoon climate.

Specimens examined: The species was discovered in the lateritic soils of Jiangxi and Guangxi provinces in southern China (25°6′0″ N–115°42′0″ E and 25°21′36″ N–115°22′48″ E, 25°4′48″ N–111°18′36″ E); collected by J.L. Zhang in April 2018. Holotype: Deposited at the China General Microbiological Culture Collection Center (CGMCC, No. 23293) and the Center for Subtropical Arbuscular Mycorrhizal Fungi Culture Collection (CSMC, No. CSMC-JX1).

Molecular and phylogenetic analyses: The obtained SSU-ITS-LSU rDNA sequences were uploaded to NCBI and subjected to BLAST comparison. In the BLASTn analysis, the most closely related species to *A. citrusnsis* was *A. papillosa*: LN884303 (97.20%), LN884302 (97.20%), LN884301 (97.05%) [48], and uncultured *Acaulospora* (97.26%) (KF849639) [68,69]. Phylogenetic analyses of the 18S-ITS1-5.8S-ITS2-28S region firmly place *A. citrusnsis* in a branch of the genus *Acaulospora*, containing *A. papillosa*, *A. favopapillosa*, *A. mellea,* and *A. rugosa*, and clearly separate this species from its closest relatives and from each other using maximum likelihood analyses and Bayesian analyses (Figure 1).

Notes: *Acaulospora citrusnsis* is a small-spored species with bright yellow color and multiple walls, which makes it distinguishable from *A. dilatata*, *A. fragilissima*, *A. gedanensis*, *A. longula*, *A. morrowae*, *A. papillosa*, *A. mellea*, *A. saccata*, and *A. trappei* though they have the same spore size. Spores of *A. mellea* have a deeper yellow color, yellow-brown walls, and usually a slightly greater diameter than *A. citrusnsis*. *A. longula* has dull, subhyaline to pale yellow spores and usually with a thinner spore wall layer 2. *Acaulospora saccata* has two spore wall layers, and the innermost layer stains deep beetroot purple in Melzer’s reagent and usually has a slightly smaller diameter than *A. citrusnsis* [47]. Spores of *A. morrowae* are bright yellow and appear to “sparkle” in reflected light and rarely have an attached hyphal terminus. Spores of *A. trappei* have a single hyaline wall, and the spore is in close proximity to the terminus. *A. papillosa* differs from *A. citrusnsis* by the presence of “small papilla” on its spore wall layer 1.

**Figure 3:**
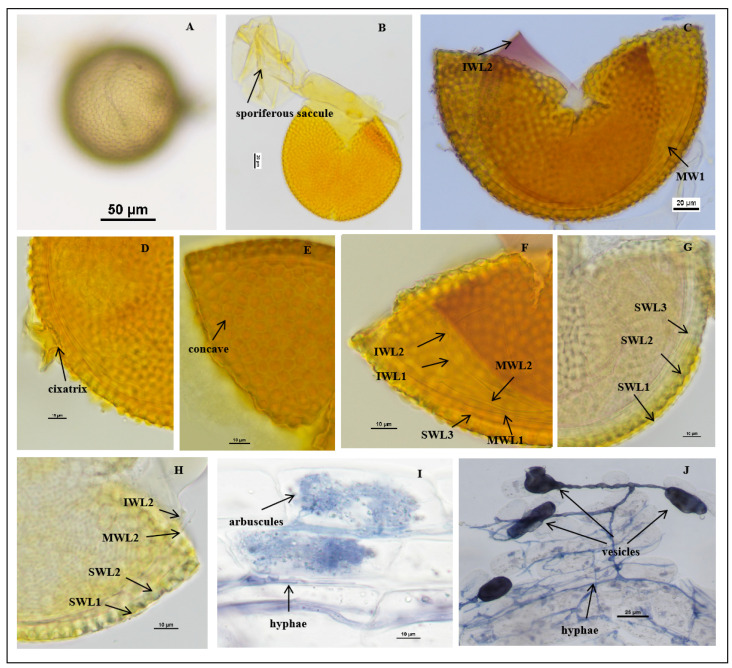
*Acaulospora guangxiensis* spores. (**A**) Intact spores in water; (**B**) crushed spore and sporiferous saccule; (**C**–**H**) spore wall layers, L1 smooth, hyaline and evanescent and completely shed; SWL2 and SWL3 laminate and similar color; in some spores, the boundaries between layers are difficult to distinguish; middle wall (MWLl–MWL2) hyaline, semiflexible bilayered tightly adherent; inner wall (IWL1–IWL3) hyaline, consists of three layers. Note: MW and IW are easily separated in PVLG, and IWL3 shows a pale pink reaction to Melzer’s reagent. (**G**,**H**) Cicatrix circular to subcircular, slightly raised collar; (**I**,**J**) mycorrhizal structures of *A. guangxiensis* in roots of *C. junos* stained in Quink ink–vinegar solution: hyphae (**J**), vesicles (**J**), and arbuscules (**I**); (**B**–**F**) in Melzer’s reagent; (**G**) in PVLG + Melzer’s reagent; (**H**) in PVLG reagent.

*Acaulospora guangxiensis* J.L.Zhang and Y.Y.Wen, sp. nov. Figure 3A–J 

MycoBank MB 856568

Description: Sporocarps unknown. Spores are formed singly in soils and laterally near the end of the proximal sporiferous saccule pedicels, pale yellow, pale yellowish brown, and some are straw-colored. The spores are spherical to sub-spherical in shape, (103.1–)122.1(–147.1) μm in diameter (n = 96) (Figure 3A,B). The spore wall consists of three walls: outer, middle, and inner walls (SW, MW, and IW). Spore wall with three layers (SWL1–3): SWL1 is hyaline and forms the surface of the spore, embedded in SWL2 layer to form a “wavy” shape, 1.5–4.1 μm thick, and is usually absent in mature spores, stained pale yellow in Melzer’s reagent (Figure 3C,F–H). SWL2 is a permanent layer that thickens initially by formation of pale yellow to pale yellowish brown, sublayers (or laminae) with polygonal concave depressions on the surface, 8.0–9.0 μm thick. The surface of the concave is mostly regular with six sides, varying in size, but regular in shape, 2.6–7.7 μm in width and 2.3–3.6 μm in depth. The ridge width between pits is 0.5–2.9 μm, and the bottom of the pit is smooth and u-shaped (Figure 3F–H). SWL3 is a hyaline layer that sometimes slightly separates from the spore wall if the spore is ruptured, with a defined boundary. It is considered to be part of the spore wall because it is usually intact (adhering to the wall) and sometimes shows evidence of being just another sublayer (lamina) of the spore wall (Figure 3F–H).

The middle wall is bi-layered (MWL1–2). The layers are tightly adherent, hyaline, of almost equal thickness, and together 0.8–1.2 µm thick (Figure 3C,F–H). Neither of them reacts in Melzer’s reagent and sometimes may produce one or more folds, giving the appearance of a bewildering array of flexible inner walls and making diagnosis difficult.

The inner wall is triple-layered (IWL1–3) and flexible hyaline. The layers are commonly tightly adherent and can be separated when the spore is ruptured (Figure 3F–H). IWL1 and IWL2 are 0.8–1.2 µm thick, with granular excresences (or “beads”) that tend to become dislodged and float away with applied pressure. IWL3 is 1.0–3.0 µm thick, stains light purplish pink to purplish red (occasionally are not colored) in Melzer’s reagent (Figure 3F–H). Cicatrix: circular to subcircular, the depression within the scar is smooth, 15.0–18.0 μm in diameter. The pore is closed by the inner laminae of L2 and L3 (Figure 3D).

Mycorrhizal associations: In the field, *A. guangxiensis* is associated with the rhizosphere of citrus *C. reticulata* (*P. trifoliata*). In monosporic cultures with the *C*. *junos* host plant, *A. guangxiensis* formed mycorrhiza with arbuscules, vesicles, and hyphae (Figure 3I,J).

Etymology: *Acaulospora guangxiensis* (Latin), referring to the site of Guangxi province in China, where this AM fungi species was recorded for the first time.

Distribution and habitat: Spores of *A. guangxiensis* were isolated from rhizosphere soil of a *citrus* orchard from Guangxi province in South China: lateritic soil and subtropical monsoon climate.

Specimens examined: The species was discovered in the rhizosphere soil of citrus plants in Guangxi Province (24°48′36″ N, 111°18′36″ E); collected by J.L. Zhang in April 2018. Holotype: Deposited at the Center for Subtropical Arbuscular Mycorrhizal Fungi Culture Collection (CSMC, No. CSMC-E25).

Molecular and phylogenetic analyses: The obtained SSU-ITS-LSU rDNA sequences were uploaded to NCBI and subjected to BLAST comparison. No sequence similarity is greater than 97%. The most closely related species to *A. guangxiensis* was *A. scrobiculata*: FR692353 (96.09%), FR692352 (95.98%), FR692354 (95.83%) [61], and *A. minuta* FR869690 (95.88%) [64].

Phylogenetic analyses of the 18S-ITS1-5.8S-ITS2-28S region firmly place *A. guangxiensis* in a branch of the strong support value (ML-BS = 92%, BPP = 1.00), containing *A. spinosa*, *A. tuberculate*, *A. minuta,* and *A. scrobiculata*, and *A. guangxiensis* was related to *A. tuberculata* and *A. spinosa* clustered together in a low support subclade (ML-BS = 95%, BPP = –) (Figure 1). Morphological and molecular evolution analyses confirmed that this species was a new species of *Acaulospora*.

Note: *Acaulospora guangxiensis* forms spores similar to those of *A. scrobiculata*. The two species are very similar in color and size, but *A. guangxiensis* differs from *A. scrobiculata* in spore wall layers. The SWL2 of *A. guangxiensis* was 8.0–9.0 μm thick, which was thicker than that of *A. scrobiculata* (4.5–7.0 μm). Moreover, the SWL2 of *A. guangxiensis* is covered with polygonal concave depressions on the surface. The concave is mostly regular with 6 sides, varying in size, but regular in shape, 2.6–7.7 μm wide and 2.3–3.6 μm deep. The ridge width between pits is 0.5–2.9 μm, and the bottom of the pit is smooth and u–u-shaped. While SWL2 of *A. scrobiculata* is covered with 0.6–2.0 μm across, 0.5–1.4 μm deep ovoid concave depressions on the surface, and some merge together to form channels 5–12 m long.

**Figure 4:**
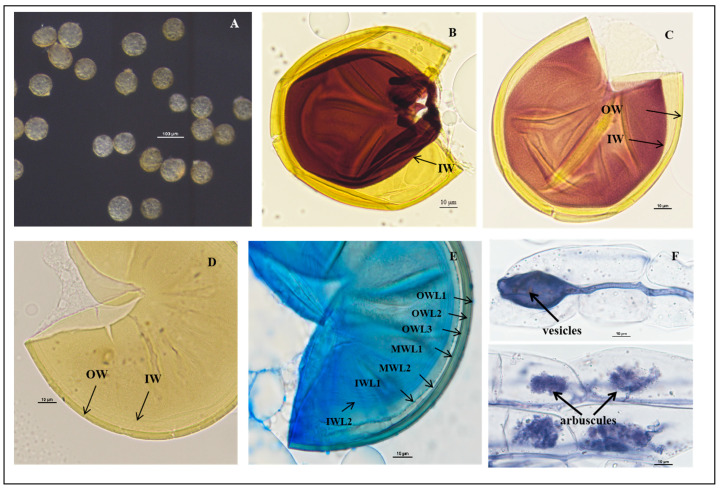
*Acaulospora jiangxiensis* spores. (**A**) Intact spores in water; (**B**–**E**) spore wall layers: SWL1 hyaline, forming the spore surface, usually absent in mature spores; SWL2 and L3 pale yellow to pale yellowish brown, permanent, laminate and similar color; in some spores, the boundaries between layers are difficult to distinguish; germinated wall hyaline consists of two layers. IWL2 shows a reaction to Melzer’s reagent; (**F**) mycorrhizal structures of *A. jiangxiensis* in roots of *C. junos* stained in Quink ink–vinegar solution: hyphae, vesicles, and arbuscules; (**B**) in Melzer’s reagent; (**C**) in PVLG + Melzer’s reagent; (**D**) in PVLG reagent; (**E**) in laetophenol cotton blue.

*Acaulospora jiangxiensis* J.L.Zhang, sp. nov. Figure 4A–F

Fungal MycoBank MB 856569

Description: Sporocarps unknown. Spores singly in soil, globose to subglobose, and the young spores are transparent and colorless, gradually mature to light yellow, occasionally oval, (78.7–)85.6(–90.0) μm in diameter, formed laterally on the sporogenous hypha (Figure 4A). No sporiferous saccule was observed in single-species culture with *Zea mays* and *C. junos*.

The spore wall consists of three walls: outer, middle, and inner walls (SW, MW, and IW). Outer spore wall is triple-layered (SWL1–3). SWL1 is hyaline, with debris on the surface of young spores, disappearing during spore maturation, (1.0–)1.2(−1.5) μm thick (Figure 4F); SWL2 is laminate, pale yellow (1.7–)2.3(−3.9) μm thick (0/0/50/10) (Figure 4B–F); SWL3 commonly adhered to L2 and can be separated after spore rupture. The middle wall (MW) is transparent and ductile with a thickness of 0.7–1.0 μm, hyaline (Figure 4). The inner wall is triple-layered (IWL1–3). The inner wall IW2 is a ductile wall, with 2 layers, IW2L1 surface particles about 1.0 μm, IW2L2 membrane wall, PVLG expansion, thickness about 1.5–3.0 μm, dyed brick red in Melzer’s reagent. Melzer’s + PVLG (1:1) reagent is light earthy red; lactic acid phenol cotton blue dyed blue; shedding marks: 8.0–10.0 μm. Inclusions: oil droplets, colorless, and transparent (Figure 4).

Mycorrhizal associations: In the field, *A. jiangxiensis* is associated with the rhizosphere of citrus *C. reticulata* (*P*. *trifoliata*). In monosporic cultures with the *C*. *junos* host plant, *A. jiangxiensis* formed mycorrhiza with arbuscules, vesicles, and hyphae (Figure 4F).

Etymology: *Acaulospora jiangxiensis* (Latin), referring to the site of Jiangxi province in China, where this AM fungi species was recorded for the first time.

Distribution and habitat: Spores of *A. jiangxiensis* were isolated from rhizosphere soil of a citrus orchard from Jiangxi province in South China: lateritic soil and subtropical monsoon climate.

Specimens examined: The species was discovered in the rhizosphere soil of citrus plants in Jiangxi Province (25°50′00″ N, 115°45′00″ E); collected by J. L Zhang in April 2018. Holotype: Deposited at the Center for Subtropical Arbuscular Mycorrhizal Fungi Culture Collection (CSMC, No. CSMC-FC-2-8).

Molecular and phylogenetic analyses: The obtained SSU-ITS-LSU rDNA sequences were uploaded to NCBI and subjected to BLAST comparison. No sequence similarity is greater than 97%. The most closely related species to *A. jiangxiensis* was *A. saccata*.

Phylogenetic analyses of the 18S-ITS1-5.8S-ITS2-28S region firmly place *A. jiangxiensis* in a branch of the genus *Acaulospora* and form a strongly supportive clade (ML-BS = 87%, BPP = 1.00) with *A. saccata* from New Caledonia (Figure 1). Morphological and molecular evolution analyses confirmed that this species was a new species of *Acaulospora*.

Note: *Acaulospora jiangxiensis* forms spores similar to *A. saccata*. The two species are very similar in color and size. *A. saccate* has two layers of spore wall, and the inner wall 2 is dyed brick red in Melzer’s reagent.

#### 3.2.2. New Record Species

**Figure 5:**
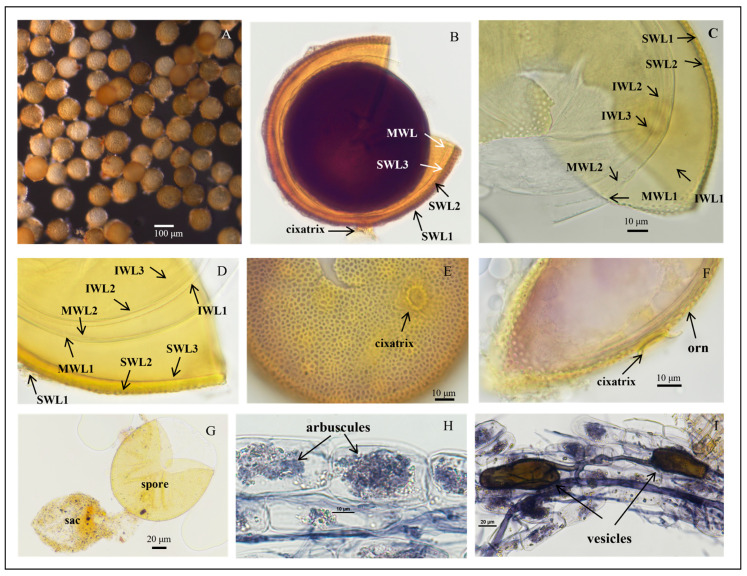
*Acaulospora herrerae* spores. (**A**) Intact spores in water; (**B**) crushed spores in Melzer’s reagent; (**C**,**D**) crushed spores in cross-view with three walls: triple-layered outer wall (SWL1–SWL3), bi-layered middle wall (MWL1–MWL2), and triple-layered inner wall (IWL1–IWL3). Outer wall with pitted ornamentation on SWL2 (orn); IWL1 with granular (“beaded”) appearance; IWL2 and IWL3 staining purple to dark purple in Melzer’s reagent; (**E**,**F**) diagnostic pitted spore surface ornamentation in planar view, showing high pit density per µm^2^, and the raised reticulate appearance; (**G**) spores with sporiferous saccules (sac) attached; spores form laterally on the saccule neck; (**H**,**I**) mycorrhizal structures of *A. herrerae* in roots of *C. junos* stained in Quink ink–vinegar solution: hyphae, vesicles, and arbuscules; (**B**) in Melzer’s reagent; (**C**,**D**,**G**) in PVLG reagent; (**E**,**F**) in PVLG + Melzer’s reagent.

*Acaulospora herrerae* Furrazola, B.T.Goto, G.A.Silva, Sieverd. and Oehl, Nova Hedwigia (2013) (Figure 5).

Description: Sporiferous saccules are 69.0–101.0 µm in diameter (Figure 5G) often detaching from mature spores, colorless, and hyaline. The pedicle usually breaks close to the base of the spore, revealing the cicatrix, which is 5.6–10.0 µm wide.

Spores forming singly in the soil are globose to subglobose, (86.3–)108.5(–127.2) μm in diameter (n = 102) or occasionally irregularly shaped; when young, spores are light yellow to yellow, becoming yellow-brown with some spores being dark yellow-brown when mature.

The spore wall consists of three walls: outer, middle, and inner walls (SW, MW, and IW). The outer wall is triple-layered (Figure 5B–D). SWL1 is hyaline, 0.8–1.2 μm thick, evanescent, and commonly absent in mature spores. SWL2 is yellow to yellowish-brown, 4.0–6.7 μm thick, lamellated, with irregularly shaped pits such as oval, round, and polygonal, typically covering the entire surface of the spore. The pits are 0.8–3.1 μm wide and 0.5–1.4 μm deep. The middle is separated by a ridge 0.3–2.5 μm wide. The spore surfaces are densely crowded with pits, leaving narrow ridges of 0.8–2.5 µm width in between each other and giving the appearance of a raised reticulum. SWL3 is hyaline, 0.5–1.0 µm thick, and often adherent to SWL2, although a slight separation occurs in some crushed spores (Figure 5C,D). The middle wall is bi-layered (MWL1–2) and smooth. The layers are tightly adherent, hyaline to pale yellow, of almost equal thickness, and together 1.1–1.7 µm thick (Figure 5C,D). The inner wall is triple-layered (IWL1–3). IWL1 is colorless and transparent, 0.7–0.9 μm thick, moniliform, and may desegregate as granular excrescences or “beads” dislodging outward with applied pressure; do not change color in Melzer’s reagent and are dyed light blue in lactic acid phenolic cotton blue reagent. IWL2 is 1.4–2.5 µm thick, expands sometimes up to 4.0 µm in PVLG. IWL3 is a very thin (<0.5 µm thick) and highly flexible layer that commonly wrinkles, showing several folds on the inner IW surface (Figure 5C,D). IWL3 is not observed in all spores, but it is frequently found. IWL2 and IWL3 stain purple to dark purple in Melzer’s reagent just after staining, and light purple in Melzer’s–PVLG (1: 1) reagent (Figure 5B); dark blue in the lactic acid phenolic cotton blue reagent; shedding mark: round or oval: 8.0–(10.33)–13.3 μm in diameter (Figure 5F). Inclusions: colorless transparent oil droplets (Figure 5F,G).

Mycorrhizal associations: In the field, *A. herrerae* is associated with the rhizosphere of Newhall navel orange (*P. trifoliata*). In monosporic cultures with the *C*. *junos* host plant, *A. herrerae* formed mycorrhiza with arbuscules and vesicles (Figure 5H,I).

Distribution and habitat: Spores of *A. herrerae* were isolated from rhizosphere soil of a *citrus* orchard from Jiangxi province in South China: lateritic soil and subtropical monsoon climate.

Specimens examined: The species was discovered in the rhizosphere soil of citrus plants in Jiangxi and Guangxi provinces (24°48′36″ N–111°18′36″ E and 25°06′00″ N–111°18′36″ E, 25°29′24″ N–115°24′00″ E); collected by J.L. Zhang in April 2018. Collected specimens deposited at the Center for Subtropical Arbuscular Mycorrhizal Fungi Culture Collection (CSMC, No. CSMC-BH2-9).

Molecular and phylogenetic analyses: The obtained SSU-ITS-LSU rDNA sequences were uploaded to NCBI and subjected to BLAST comparison, with more than 97.5% similarity to *A. herrerae*.

Phylogenetic analyses of the 18S-ITS1-5.8S-ITS2-28S region placed our discovered AM fungi firmly in a branch of the genus *Acaulospora* and formed a strongly supported clade with *A. herrerae* from Peru (ML-BS = 99%, BPP = 1.00). Morphological and molecular evolutionary analyses confirm that the species is *A. herrerae* and is found for the first time in China.

Notes: The new fungus was first reported in a calcareous soil in Eastern Cuba, and in NE Brazil, it was often found in soils with pH > 6.0 [70]. However, recently, it was also found in acidic tropical soils (pH 4.4–5.8), and it had been easily propagated in single-species cultures with a soil pH of 5.4 during the last decade. Many *Acaulospora* species have often been reported to prefer more acidic soil pH conditions for their occurrence. We assume that *A. herrerae* has a major distribution in tropical Central and South America, and that this fungus has a better adaptation to higher pH soils in tropical areas than several other *Acaulospora* species. The major morphological differences between *A. herrerae* and the fungi mentioned above are summarized as follows: *A. herrerae* produces smaller spores and more irregular pits than *A. cavernata* and *A. punctata*, respectively [63,70], and the last two species rarely show the appearance of a raised reticule. *A*. *excavata* [71] produces larger spores and much larger pits than *A. herrerae*, and *A. lacunose* [72] forms spores larger than those of *A. herrerae* (98–186 µm vs. 50–112 µm). Also, *A. lacunosa* shows a reddish-yellow color, and its ornamentation differs from the one described for *A. herrerae* as spores of *A. lacunosa* are ornamented with highly irregular saucer-shaped pits 0.2–3.0 × 0.2–6.0 µm broad, 0.2–2.0 µm deep, and highly variable in number, showing also cone-shaped raised edges [72]. The spore sizes of *A. paulinae* [73] and *A. nivalis* [62] overlap with those of *A. herrerae* as well as their pit diameter. However, *A. paulinae* and *A. nivalis* pits were not described to show a raised reticule, respectively. Spores of *A. scrobiculata* [74] are much bigger (up to 240 µm) than those of *A. herrerae*. Also, *A. scrobiculata* spores are evenly pitted with circular, elliptical, or linear to y-shaped depressions 1.0–1.5 × 1.0–3.0 µm, and they also do, like minutely pitted *A. minuta*, not have a raised reticule [61,74]. Spores of *A. sieverdingii* are much lighter in color and also lack a reticulum. Finally, spores of *A. kentinensis* are formed within the neck of sporiferous saccules, while spores of *A. herrerae* are always formed laterally on the neck of their saccules. Spores of *A. kentinensis* also never form a reticulum. Moreover, pit density of *A. herrerae* is high (187–212 pits 500 µm^−2^; mean = 199), while the pits of *A. kentinensis* isolate SM-71 from Taiwan conserved in sodium azide solution (courtesy of Dr. C. G. Wu) are less numerous (30–43 pits 500 µm^−2^; mean = 36), and a similar Cuban isolate of *A. kentinensis* (CCHMA accession IES-56) had 44–68 pits/500 µm^−2^ (mean = 58). Pit density analyses, so far rarely performed on AM fungi species, might indeed be useful in the future for the identification of ornamented glomeromycotan species, but this characteristic will need to be investigated in more detail for all of these species.

## 4. Discussion

Arbuscular mycorrhizal fungi are widely found in soils around the world, and their biological and functional diversity plays an important role in agroecosystems. As of 2017, about 150 species of AM fungi have been discovered and reported in China, including 13 new species [75]. In this study, more than 1500 single spores were extracted from 90 soil samples, which were collected from Jiangxi and Guangxi provinces. According to the morphological characteristics and phylogenetic analysis, three new species, viz. *A. citrusnsis*, *A. guangxiensis*, *A. jiangxiensis*, and a newly recorded species from China, viz. *A. herrerae*, were identified.

In appearance, the newly discovered AM fungi *A. citrusnsis*, *A. guangxiensis*, and *A. jiangxiensis* are easily distinguished from other species found in the genus *Acaulospora* by their spore color, size, and spore characteristics. The spores of new species *A. citrusnsis* have a similar size to *A. saccata*, *A. longula*, and *A. apillosa*. The spores of *A. saccata and A. longula* are very similar in color and size [47,76]. However, their spore characteristics still differ in detail. In addition, the spores of new species *A. citrusnsis* and *A. longula* are essentially identical in appearance, but they differ in the number of inner wall layers (IW) and their reaction in Melzer’s reagent: *A. longula* has a single inner wall layer, which appears pale purple in Melzer’s reagent [76], whereas *A*. *citrusnsis* has two inner wall layers, which appear deep purplish-red to brownish-red in Melzer’s reagent. *A. saccata* possesses a bilayered spore wall, with the inner layer being unilamellar. *A. jiangxiensis* consists of three layers, with the inner layer (inner wall 1) comprising two distinct sublayers. Other features clearly separate *A*. *citrusnsis* from *A. saccata*. The IWL2 in *A. saccata* is plastic and stains purple in Melzer’s reagent, whereas *A. citrusnsis* is flexible, does not swell in PVLG (i.e., it is not plastic), and stains deep purplish red to brownish red in Melzer’s reagent. The spore surface of *A. papillosa* is rough and covered with fine papillae, while *A*. *citrusnsis* is smooth. Based on the ornamentation pattern of the spores, *A. guangxiensis* may be confused with other species with spiny projections, such as *A. spinosa*, *A. tuberculata*, and *A. scrobiculata*, but from the color and size of the spores, *A. guangxiensis*, *A. spinosa,* and *A. tuberculata* are easy to distinguish. The spores of *A. spinosa* were dull yellowish brown to dark reddish brown, with dimensions of 100–298 × 100–335 μm; while the spores of *A. tuberculata* were dark honey brown to reddish black, with dimensions of 255–327 × 255–340 μm [60].

The spores of *A. guangxiensis* are very similar to those of *A. scrobiculata* in color and size, but they differ in spore wall layers. The SWL2 of *A. guangxiensis* was 8.0–9.0 μm thick, covered with a regularly shaped polygonal depression of varying size, ranging from 2.6–7.7 μm wide to 2.3–3.6 μm deep and the ridge between the pits is 0.5–2.9 μm wide, the bottom of the pits is smooth and U-shaped, while *A. scrobiculata* is 4.5–7.0 μm thick, and ornamented with evenly distributed pits: circular, ellipsoidal, oblong, triangular, Y-shaped to irregular [25].

Under the dissecting microscope, *A. jiangxiensis* may be easily confused with other small, light-colored species such as *A. longula*, *A. delicata*, *A. saccata*, and the newly discovered *A. citrusnsis*. These species share similar characteristics in terms of size, color, and smooth spore walls. The inner wall layer (IW) of *A. longula* consists of a single layer and appears light purple in Melzer’s reagent. In contrast, the inner wall layer (IW) of *A. jiangxiensis* comprises two layers and exhibits a brick-red color in Melzer’s reagent. These distinct differences in layer structure and reagent reaction facilitate easy differentiation between *A. longula* and *A. jiangxiensis*. The spore walls of *A. delicata* and *A. saccata* consist of only two layers, whereas the spore wall of *A. jiangxiensis* has three distinct layers. The spore morphology of *A. citrusnsis* and *A. jiangxiensis* is quite similar: Both species produce colorless and transparent spores when young, which turn pale yellow at maturity. However, a key difference lies in the surface of the first spore wall layer (SWL1). In *A. citrusnsis*, SWL1 is typically covered with debris and measures 0.7–1.0 μm in thickness, while in *A. jiangxiensis*, this layer remains free of debris. Additionally, these two species are well-distinguished from each other on the phylogenetic tree.

According to the phylogenetic analysis, the phylogenetic tree can be divided into five clades: Clade I includes seven species: *A. rugosa*, *A. mellea*, *A. favopapillosa*, *A. citrusnsis*, *A. papillosa*, *A. jiangxiensis*, and *A. saccata*. Notably, these species are characterized by spore surfaces that are either smooth or rough, with small spores lacking permanent ornamentation. Clade II includes six species: *A. baetica*, *A. ignota*, *A. nivalis*, *A. sieverdingii*, *A. punctata*, and *A. cavernata*. The spore wall ornamentation of five of these species features depressions, while *A. ignota* has spores with warts or flattened elevations [54,58,62,63,64,77]. Clade III comprises five species: *A. herrerae*, *A. excavata*, *A. aspera*, *A. mendoncae,* and *A. spinosissima*. The first three species have spore wall ornamentations characterized by depressions, while the latter two species feature projections; *A. guangxiensis* forms Clade IV along with *A. minuta*, *A. scrobiculata*, *A. spinosa*, and *A. tuberculata*. The spores of these five species exhibit distinct ornamentations: those of *A. guangxiensis*, *A. minuta*, and *A. scrobiculata* feature depressions, whereas the spore walls of *A. spinosa* and *A. tuberculata* display projections [61,78,79]. Clade V includes *A. brasiliensis*, *A. gedanensis*, *A. tortuosa*, and *A. alpina*. Among these species, only the spores of *A. gedanensis* lack ornamentations. The spores of the other three species are ornamented: *A. tortuosa* features projections, while *A. brasiliensis* and *A. alpina* exhibit depressions [52,56,66]. Among these 5 clades, only the species in clade I conform to the principle of morphological characteristics, where spores are clustered together without ornamentations. The other four clades do not adhere to this principle. This phenomenon indicates that the ornamentations of the spore do not follow the constructed historical evolution, thus confirming that spore ornamentations should not be used for phylogenetic inference but are only applicable for the morphological description of the species [25].

Many species of the genus *Acaulospora* have been reported to breed in acidic soils [69]. In this study, all four AM fungi described were found in acidic red soils with a pH below 6.0. Specifically, *A. citrusnsis* was found in soils with a pH range of 4.03–4.73, *A. guangxiensis* in soils with a pH of 5.23, and *A. jiangxiensis* in soils with a pH of 4.26. *A. herrerae* was discovered in soils with a pH range of 4.27–5.93. Notably, this species has also been reported in northeastern Brazil in soils with a pH greater than 6.0, indicating its strong adaptability to higher pH conditions. This evidence suggests that while most *Acaulospora* species prefer acidic environments, some, like *A. herrerae*, can tolerate and thrive in more alkaline soils.

In summary, the morphological and molecular analyses conducted in this study provide robust evidence that the described AM fungi species are indeed new to science. These are the first AM fungi newly described from China and have thus far been detected only in soils from subtropical monsoon climates and lateritic soils. Further research is necessary to determine whether these species are restricted to this specific environment and whether they form associations with particular plant hosts.

## Figures and Tables

**Table 1:** Isolation sites of the *Acaulospora* genus with geographic data, soil pH, organic carbon, and available phosphorus.

AM Fungi Species	Location	Province	Citrus Varieties	Geographic Position	Mean Annual Temperature (°C)	Mean Annual Precipitation (mm)	pH	Organic C (g/kg)	Available P (mg/kg)	NH_4_^+^-N (mg/kg)	NO_3_^−^-N (mg/kg)
*Acaulospora citrusnsis*	Xunwu County	Jiangxi	Newhall navel orange	25°6′0″ N 115°42′0″ E	19.00	1650.30	4.46	24.24	90.70	19.16	43.58
	Banshi Town, Anyuan County	Jiangxi	Hamlin sweet orange	25°21′36″ N 115°22′48″ E	18.60	1670.00	4.03	24.20	361.92	39.51	25.41
	Gepo Town, Fuchuan County	Guangxi	Sugar oranges	25°4′48″ N 111°18′36″ E	18.90	1420.60	4.73	29.33	120.40	20.24	42.95
*Acaulospora guangxiensis*	Mailing Town, Fuchuan County	Guangxi	Wenzhou mandarin	24°48′36″ N 111°18′36″ E	18.00	1374.50	5.23	22.45	44.30	3.89	5.11
*Acaulospora herrerae*	Mailing Town, Fuchuan County	Guangxi	Wenzhou mandarin	24°48′36″ N 111°18′36″ E	18.00	1374.50	5.23	22.45	44.30	3.89	5.11
	Fuyang Town, Fuchuan County	Guangxi	Newhall navel orange	25°06′00″ N 111°18′36″ E	19.20	1721.00	5.93	24.58	167.48	5.41	8.44
	Longbu Town, Anyuan County	Jiangxi	Newhall navel orange	25°29′24″ N 115°24′00″ E	18.00	1584.00	4.27	18.67	214.33	4.58	2.44
*Acaulospora jiangxiensis*	Fucuo Town, Anyuan County	Jiangxi	Hamlin sweet orange	25°50′00″ N 115°45′00″ E	17.00	1594.00	4.26	16.67	174.88	8.50	5.69

**Table 2:** Sequences used in phylogenetic analyses.

Number	Species	Origin	GenBan Accession Number		Reference
			SSU-ITS-LSU	LSU	
1	*Acaulospora alpina*	UK	FR681929		[52]
2	*Acaulospora alpina*	UK	FR681930		[52]
3	*Acaulospora aspera*	Pe	MN081000		[53]
4	*Acaulospora aspera*	Pe	MN081001		[53]
5	*Acaulospora baetica*	ES	LN811001		[54]
6	*Acaulospora baetica*	ES	LN811002		[54]
8	*Acaulospora brasiliensis*	UK	FN825904		[52]
9	*Acaulospora brasiliensis*	UK	FN825906		[52]
10	*Acaulospora cavernata*	UK	FR692347		GenBank
11	*Acaulospora cavernata*	UK	FR692348		GenBank
12	*Acaulospora citrusnsis*	CN	OL757539		This study
13	*Acaulospora citrusnsis*	CN	OL757540		This study
14	*Acaulospora citrusnsis*	CN	OL757541		This study
15	*Acaulospora citrusnsis*	CN	OL757542		This study
16	*Acaulospora excavata*	Pe	OK356203		[55]
17	*Acaulospora excavata*	Pe	OK356204		[55]
18	*Acaulospora favopapillosa*	Pe	OK360960		[55]
19	*Acaulospora favopapillosa*	Pe	OK360963		[55]
20	*Acaulospora favopapillosa*	Pe	OK360964		[55]
21	*Acaulospora fragilissima*	Fr	KY362431		[47]
22	*Acaulospora fragilissima*	Fr	KY362432		[47]
23	*Acaulospora gedanensis*	PL	OR669015		[56]
24	*Acaulospora gedanensis*	PL	OR669019		[56]
25	*Acaulospora guangxiensis*	CN	PQ461066		This study
26	*Acaulospora guangxiensis*	CN	PQ461067		This study
27	*Acaulospora guangxiensis*	CN	PQ461068		This study
28	*Acaulospora guangxiensis*	CN	PQ461069		This study
29	*Acaulospora guangxiensis*	CN	PQ461070		This study
30	*Acaulospora herrerae*	Pe	MZ191547		[57]
31	*Acaulospora herrerae*	Pe	MZ191548		[57]
32	*Acaulospora herrerae*	CN	PQ461061		This study
33	*Acaulospora herrerae*	CN	PQ461062		This study
34	*Acaulospora herrerae*	CN	PQ461063		This study
35	*Acaulospora herrerae*	CN	PQ461064		This study
36	*Acaulospora herrerae*	CN	PQ461065		This study
37	*Acaulospora ignota*	BR	KP191467		[58]
38	*Acaulospora ignota*	BR	KP191471		[58]
39	*Acaulospora lacunosa*	PT	KP756427		[59]
40	*Acaulospora lacunosa*	PT	KP756430		[59]
41	*Acaulospora mellea*	CN	JF439091		GenBank
42	*Acaulospora mellea*	CN	JF439092		GenBank
43	*Acaulospora mendoncae*	BR	OK392599		[60]
44	*Acaulospora mendoncae*	BR	OK392604		[60]
45	*Acaulospora minuta*	BJ	FR821673		[61]
46	*Acaulospora minuta*	BJ	FR821674		[61]
47	*Acaulospora nivalis*	CH	HE603643		[62]
48	*Acaulospora nivalis*	CH	HE603644		[62]
49	*Acaulospora papillosa*	BR	LN884302		[48]
50	*Acaulospora papillosa*	BR	LN884303		[48]
51	*Acaulospora punctata*	CH	FR846383		[63]
52	*Acaulospora punctata*	CH	FR846384		[63]
53	*Acaulospora rugosa*	NO	LN881564		[48]
54	*Acaulospora rugosa*	NO	LN881565		[48]
55	*Acaulospora saccata*	NC	KY362428		[47]
56	*Acaulospora saccata*	NC	KY362429		[47]
57	*Acaulospora saccata*	NC	KY362430		[47]
58	*Acaulospora jiangxiensis*	CN	PQ461057		This study
59	*Acaulospora jiangxiensis*	CN	PQ461060		This study
60	*Acaulospora jiangxiensis*	CN	PQ461058		This study
61	*Acaulospora jiangxiensis*	CN	PQ461059		This study
62	*Acaulospora scrobiculata*	BJ	FR692349		[64]
63	*Acaulospora scrobiculata*	BJ	FR692351		[64]
64	*Acaulospora sieverdingii*	CH	FM876792		[64]
65	*Acaulospora sieverdingii*	CH	FM876793		[64]
66	*Acaulospora spinosa*	US	FR750154		GenBank
67	*Acaulospora spinosa*	US	FR750153		GenBank
68	*Acaulospora spinosissima*	BJ	HG422732		[65]
69	*Acaulospora spinosissima*	BJ	HG422733		[65]
70	*Acaulospora tortuosa*	ES		HF567933	[66]
71	*Acaulospora tortuosa*	ES		HF567937	[66]
72	*Sacculospora baltica*	PL	KX355819		[67]
73	*Sacculospora baltica*	PL	KX355821		[67]
74	*Sacculospora felinovii*	IN	KX345942		[67]
75	*Sacculospora felinovii*	IN	KX345939		[67]

## Data Availability

The original contributions presented in this study are included in the article. Further inquiries can be directed to the corresponding author.
